# Spontaneous Otoacoustic Emissions in *Tecta^Y1870C/+^* Mice Reflect Changes in Cochlear Amplification and How It Is Controlled by the Tectorial Membrane

**DOI:** 10.1523/ENEURO.0314-18.2018

**Published:** 2018-12-26

**Authors:** Mary Ann Cheatham, Yingjie Zhou, Richard J. Goodyear, Peter Dallos, Guy P. Richardson

**Affiliations:** 1 Knowles Hearing Center; 2 Roxelyn and Richard Pepper Department of Communication Sciences and Disorders, Northwestern University, Evanston IL 60208; 3 Sussex Neuroscience, School of Life Sciences, University of Sussex, Falmer, Brighton, BN1 9QG, United Kingdom

**Keywords:** cochlear amplifier, harmonic distortion, outer hair cells, spontaneous otoacoustic emissions, tectorial membrane

## Abstract

Spontaneous otoacoustic emissions (SOAEs) recorded from the ear canal in the absence of sound reflect cochlear amplification, an outer hair cell (OHC) process required for the extraordinary sensitivity and frequency selectivity of mammalian hearing. Although wild-type mice rarely emit, those with mutations that influence the tectorial membrane (TM) show an incidence of SOAEs similar to that in humans. In this report, we characterized mice with a missense mutation in *Tecta,* a gene required for the formation of the striated-sheet matrix within the core of the TM. Mice heterozygous for the Y1870C mutation (*Tecta^Y1870C/+^*) are prolific emitters, despite a moderate hearing loss. Additionally, Kimura’s membrane, into which the OHC stereocilia insert, separates from the main body of the TM, except at apical cochlear locations. Multimodal SOAEs are also observed in *Tecta^Y1870C/+^* mice where energy is present at frequencies that are integer multiples of a lower-frequency SOAE (the primary). Second-harmonic SOAEs, at twice the frequency of a lower-frequency primary, are the most frequently observed. These secondary SOAEs are found in spatial regions where stimulus-evoked OAEs are small or in the noise floor. Introduction of high-level suppressors just above the primary SOAE frequency reduce or eliminate both primary and second-harmonic SOAEs. In contrast, second-harmonic SOAEs are not affected by suppressors, either above or below the second-harmonic SOAE frequency, even when they are much larger in amplitude. Hence, second-harmonic SOAEs do not appear to be spatially separated from their primaries, a finding that has implications for cochlear mechanics and the consequences of changes to TM structure.

## Significance Statement

Aspects of cochlear function can be observed noninvasively by placing a sensitive microphone in the ear canal and recording stimulus-evoked otoacoustic emissions (OAEs). These responses provide information about the operation of the outer hair cells (OHCs), which are required for sensitivity and frequency selectivity, the hallmarks of normal cochlear operation in mammals. In addition to stimulus-related OAEs, spontaneous OAEs are frequently observed in humans as narrow-band signals in the absence of sound. Although wild-type mice are rarely spontaneous emitters, those with an altered tectorial membrane, the accessory structure to which the OHC stereocilia are attached, exhibit a dramatic increase in these phenomena. Determining the underlying basis for this phenotype has the potential to reveal important aspects of cochlear function.

## Introduction

In the mammalian cochlea, inner hair cells (IHCs) are the primary sensory receptors and are connected to the vast majority of auditory nerve fibers. Although the more numerous outer hair cells (OHCs) receive <10% of the afferent innervation, most of the efferents descending from the medial olivocochlear complex synapse with OHCs (Spoendlin, 1985) and modulate their function by adjusting a tightly coupled feedback loop. The latter is thought to include the OHCs, the Deiters’ cells that link the OHCs to the underlying basilar membrane (BM), the reticular lamina that interconnects the apices of all hair cells, and the tectorial membrane (TM) that overlies the organ of Corti and into which the tallest OHC stereocilia are embedded (Dallos and Fakler, 2002; Hudspeth, 2014). This feedback system produces cochlear amplification and relies on prestin-based OHC electromotility (Dallos et al., 2008).

**Figure 1.**
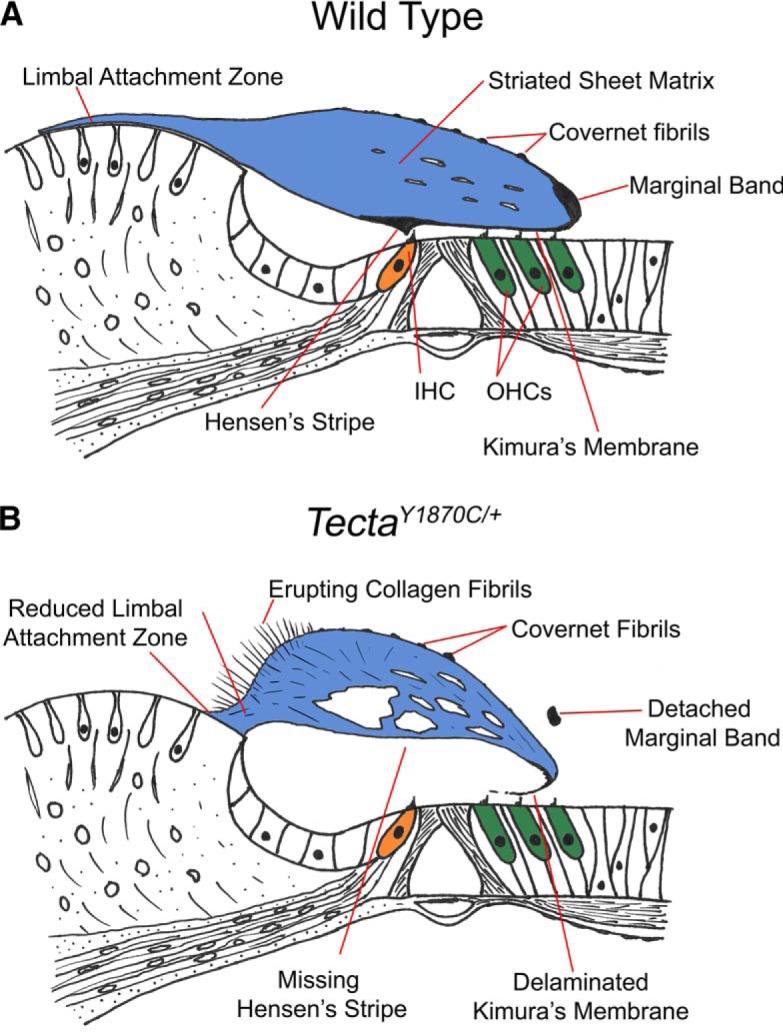
A schematic representing tectorial membrane anatomy. ***A***, The WT mouse organ of Corti. ***B***, The *Tecta^Y1870C/+^*mouse organ of Corti. The drawings are based on anatomical results published by Legan et al. (2005).

The TM extends medially from the limbal zone and terminates laterally as a thickening referred to as the marginal band (Fig. 1*A*). Several noncollagenous proteins are associated with the striated-sheet matrix within which the larger collagen fibers are embedded. These include CEACAM16, as well as α-tectorin (TECTA) and β-tectorin (TECTB; Goodyear and Richardson, 2018). In addition to the matrix, a covernet is located on the upper surface. In the basal half of the mouse cochlea, Hensen’s stripe presents as a ridge projecting down from the lower surface of the membrane just medial to the IHC stereocilia (Goodyear and Richardson, 2018). Finally, a thickening along the lower surface was originally described by Hardesty (1908), who was able to better control the histologic artifacts that plagued early efforts to examine this structure. Subsequently, Kimura (1966) showed that the tallest OHC stereocilia are inserted into this part of the TM, and thus this region has been referred to as Kimura’s membrane.

At least 33 mutations in *TECTA* are associated with both dominant (DFNA8/12) and recessive (DFNB21) forms of human hereditary deafness (Hildebrand et al., 2011). Because of this connection to hearing loss, mutants have been created for some of the human mutations in the genes that encode proteins required for the formation of the striated-sheet matrix. In mice lacking *Tecta*, hearing loss is present across frequency, consistent with loss of amplification and detachment of the TM in its entirety from the organ of Corti (Legan et al., 2000). In contrast, *Tecta^Y1870C/+^*mice heterozygous for a missense mutation (c.5609A>G, p.Tyr1870Cys) that causes human hereditary deafness (Legan et al., 2005), exhibit a semidominant phenotype. In this mutant (Fig. 1*B*), the limbal attachment is reduced, Kimura’s membrane separates from the main body of the TM while maintaining contact with the OHC stereocilia, the marginal band and Hensen’s stripe are absent, and collagen fibrils erupt from the upper surface of the membrane. Although the striated-sheet matrix remains near normal in the lateral hair cell region, large holes are prominent throughout (Legan et al., 2005). Despite these changes, the distortion product otoacoustic emissions (DPOAEs) are reduced to a lesser degree than the compound action potentials, which show large threshold shifts.

Our previous work on various TM mutants showed that several exhibited spontaneous otoacoustic emissions (SOAEs), phenomena associated with cochlear amplification and recorded in the ear canal in the absence of acoustic stimulation. SOAEs, as well as stimulus frequency otoacoustic emissions (SFOAEs), are considered by some to originate through a mechanism of coherent reflection, while DPOAEs are dominated by contributions from nonlinear distortion sources (Shera and Guinan, 1999). Although relatively common in humans (Probst et al., 1986), we observed spontaneous phenomena in only ∼6% of our control animals over the ∼10 years that we have been making these recordings. It was, therefore, surprising that 70% of *Ceacam16^βgal/βgal^* mice lacking CEACAM16 that were <8 weeks of age (67 of 95 homozygotes) produced SOAEs (Cheatham et al., 2014). In the absence of CEACAM16, the striated-sheet matrix does not form, Hensen’s stripe is absent and large holes appear, especially in the apical portion of the TM. Furthermore, 79% of *Otoa^EGFP/EGFP^* mice (34 of 43 homozygotes) lacking OTOANCORIN (OTOA), in which the TM is detached from the limbus, also produce SOAEs (Cheatham et al., 2016). Because of the human hearing problems associated with TM defects and the unusual phenotype discovered in *Ceacam16^βgal/βgal^* and *Otoa^EGFP/EGFP^* mice, we examined emissions and auditory brainstem responses (ABRs) in *Tecta^Y1870C/+^* mice and their wild-type (WT) controls. We also further defined the anatomical changes at various positions along the cochlear partition to gain further insights into the active amplification process and how it is controlled.

## Materials and Methods

### Animals

*Tecta^Y1870C^*mice were on a mixed, variable B6/129S background and were initially acquired from the Freeman Laboratory at Massachusetts Institute of Technology (Cambridge, MA; Sellon et al., 2014). Genotyping was outsourced to Transnetyx. Young male and female mice were tested using OAEs and ABRs between 3 and 9 weeks of age. Animals were excluded from the study if they showed evidence of ear canal anomalies such as extensive wax or inflammation, which was not common. It should also be understood that not all mice were tested with all measures as this can require several hours of testing and multiple doses of anesthesia. This is the reason for the varying numbers of animals in the different plots. All procedures were approved by the Northwestern University Institutional Animal Care and Use Committee, the Animal Welfare and Ethical Review Board at the University of Sussex, and by the National Institute on Deafness and Other Communication Disorders, and were performed in accordance with the UK Animals (Scientific Procedures) Act.

### Emission measurements

Following a pinna reflex test, the mouse was anesthetized using ketamine (120 mg/kg, i.p.) and xylazine (10 mg/kg, i.p.), with supplemental injections provided as needed. The animal was then moved into a sound isolation booth and placed on a heating pad. A custom emission probe containing a sensitive microphone (FG-3652-CX, Knowles Electronics, Itasca, IL) was inserted into the ear canal to form a tight seal. After the sound calibration was performed using SysRes (Neely and Stevenson, 1992), the noise floor in quiet was sampled for 3.8 min by performing a spectral average of 40 samples of the canal pressure into a 524,288 point buffer (Cheatham et al., 2014). A fast Fourier transform was performed on the time wave form with a resolution of 96,000/524,288 = 0.183 Hz. The spectrum was then smoothed before integrating energy into windows of 93 Hz. SOAEs <0 dB SPL were not used for analysis. The DPOAEs were generated using two primary stimulus tones, f1 and f2, with a frequency ratio f2/f1 = 1.2. Iso-input functions (or DP-grams) were measured for 21 f2 frequencies ranging from 3 to 47 kHz (step size increasing with f2 frequency) and for the level of f1 (L1) = 50 dB SPL and for the level of f2 (L2) = 35 dB SPL, as well as for L1 = L2 = 70 dB SPL. All signals were generated by using a 24-bit sound card (Card Deluxe) with a sampling rate of 96 kHz. Input–output functions were also acquired at f2 = 12 and f2 = 27 kHz to obtain a threshold, which was defined as the level of f1 required to generate a DPOAE of 0 dB SPL. This approach is similar to that used by the Liberman group (Maison et al., 2010). For these functions, L1 was 10 dB higher than that for L2, and stimuli increased in steps of 5–10 dB. Stimulus delivery and data acquisition were controlled using Emav (Neely and Liu, 1994). SFOAEs were measured between 4 and 38 kHz (step size, 1.172 kHz) using a single probe/suppressor level (50/75 dB SPL, respectively) combination. Probe response was measured alone and in the presence of a suppressor placed 86 Hz below probe frequency, which results in near-complete suppression of the emission (Brass and Kemp, 1993; Keefe et al., 2008). Subtracting the probe response vectors with and without the suppressor provides the SFOAE residual (Dreisbach et al., 1998; Siegel et al., 2001), which corresponds to the total SFOAE amplitude when suppression is complete. For the SOAE suppression experiments, a function generator was used to produce a sinusoid of the desired frequency. The magnitude of this external tone (T_ext_), recorded in the ear canal, was computed in the same way as that for the SOAE. The external tones were also measured in a tubing coupler with a volume that was similar to the mouse ear canal with emission probe inserted. These measurements allowed us to learn whether presentation of the external tone generated distortion in the sound. The latter was not observed for the suppressor levels reported here. Further details are provided in previous publications (Cheatham et al., 2014, 2016).

### Measurements of auditory brainstem responses

To assay the impact of TM changes on the input to the IHC stereocilia and ultimately on the output of the cochlea, ABR thresholds (mean ± SEM) were acquired at 12 and 27 kHz, the same f2 frequencies where DPOAE growth functions were also recorded. Following the emission measurements, the mice were outfitted with three subdermal needle electrodes to collect the evoked potentials. Recordings were made at the mastoid, vertex, and also at the shoulder on the side opposite to the mastoid electrode. Only a small amount of tissue was in contact with the vertex and mastoid electrodes to minimize the pickup area (Shaheen et al., 2015). Outputs of the mastoid and vertex electrodes were measured relative to the indifferent electrode in the shoulder region. These responses were differentiated, amplified 1000×, and bandpass filtered (0.3–2.0 kHz). Tone-burst stimuli were 10 ms in duration, including the 1 ms rise/fall times. Signal levels were decreased in 5 or 10 dB steps, and responses were averaged 3000 times to determine the level at which all ABR waves I–IV disappeared into the noise floor. Because the pinna was intact, the speculum was placed at the entrance to the ear canal in a quasi-free-field arrangement. SPLs were determined off-line using a real pinna coupler calibration. In this procedure, a one-eighth inch B&K condenser microphone was placed at the position of the tympanic membrane, which had been removed (Pearce et al., 2001).

### Statistical analyses

Data are reported as the means ± SEM. Some statistical comparisons were obtained using a two-tailed Student’s *t* test and assuming unequal variance between samples (Excel, Microsoft). However, see Figure 3 for results in which we performed a two-way ANOVA using a between-group factor of mouse genotype and a within-group factor of f2 frequency (see Fig. 3*A*) or stimulus level (see Fig. 3*C*,*D*). The results of Bonferroni-corrected *post hoc t* tests are also provided. Finally, a linear regression was also performed using a least-squares fitting procedure for the data plotted in Figure 5*B* [Mathematica (Wolfram.com)].

### Morphologic analysis

Following the killing of the mice, the cochleae were fixed in glutaraldehyde by immersion for ∼2 h and then postfixed in 1% OsO_4_ for ∼1 h. Samples were then decalcified, dehydrated, and embedded in epoxy resin. The 1 μm sections were obtained parallel to the modiolar axis of the cochlea such that the ∼4, 8, 20, and 40 kHz locations were obtained in a single section. These locations were designated using the Liberman Laboratory Protocol (Liberman et al., 2015). Sections were then stained with 1% toluidine blue. Further details can be obtained from previous publications (Legan et al., 2005; Cheatham et al., 2014).

## Results

### *Tecta^Y1870C/+^*mice produce numerous SOAEs although thresholds are elevated

**Figure 2.**
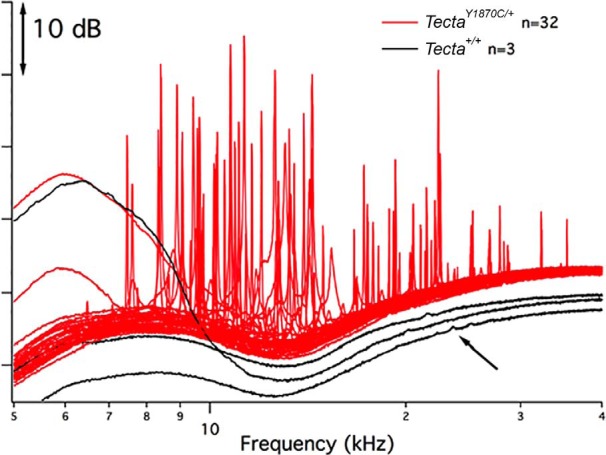
SOAE spectra. WT controls with SOAEs are plotted in black; the *Tecta^Y1870C/+^*mice are in red. To reduce overlap, the traces are arbitrarily shifted vertically.

Inasmuch as previously characterized TM mutants lacking either CEACAM16 (Cheatham et al., 2014) or OTOA (Cheatham et al., 2016) displayed an increase in SOAEs, we initially assayed for this type of emission, as shown in Figure 2. Here SOAE spectra are provided for 3 of the 20 wild-type *Tecta^+/+^* mice that had recordable SOAEs and for 32 of the 51 heterozygous *Tecta^Y1870C/+^*mice with SOAEs. Notice the striking difference between heterozygous (Fig. 2, red) and WT (Fig. 2, black) mice as to the size and the propensity to produce SOAEs. Although only 6.1% of all WT mice (16 of 262) we have studied independent of genetic background show this behavior, 62.7% of *Tecta^Y1870C/+^*mice (32 of 51) are spontaneous emitters. In this mutant, SOAEs are generated over a wide frequency range, and the average (±SEM) SOAE frequency (15.4 ± 0.8 kHz; *n* = 203) is lower than that in WT mice (22.3 ± 0.9 kHz *n* = 16; *p* < 0.01). These mutants can also generate large SOAEs. Although some approach 50 dB SPL in magnitude, the average mutant SOAE magnitude was 18.3 ± 0.3 dB SPL (*n* = 203). In contrast, the average WT magnitude was 14.2 ± 1.4 dB SPL for the small percentage of WT animals with SOAEs (*n* = 16), which is statistically lower than in heterozygotes (*p* < 0.01). In fact, the largest SOAE ever recorded in WT mice was 24.5 dB SPL. In addition, *Tecta^Y1870C/+^*mice produced an average of 6.3 simultaneous SOAEs per ear. This is nearly double that in any other mutant or in any WT control, where an average of 2.1 SOAEs are recorded in a given ear.

**Figure 3.**
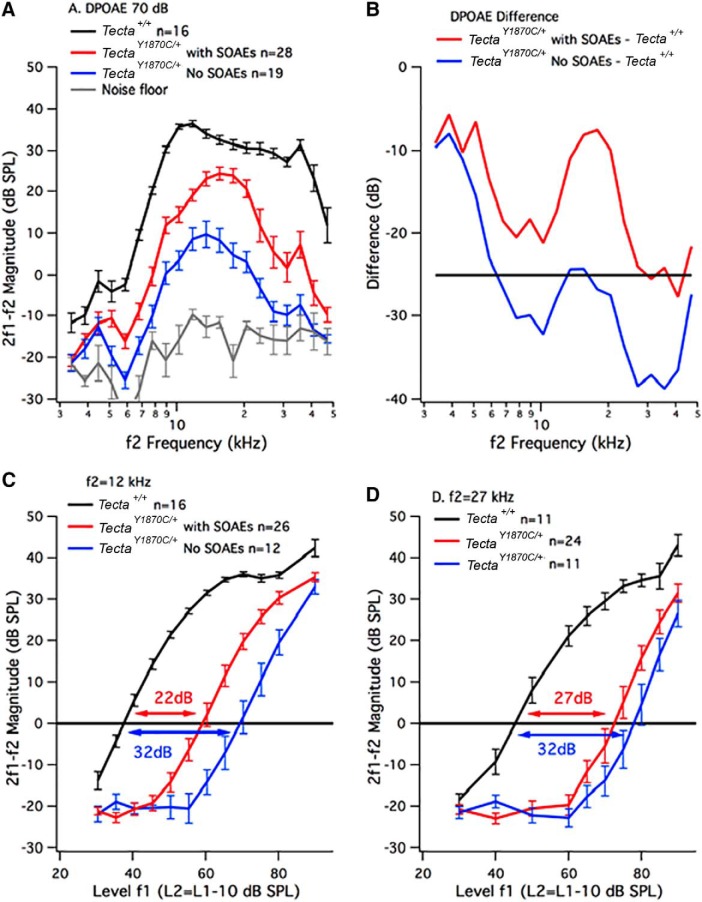
DPOAEs. ***A***, Average (±SEM) iso-input functions (DP-grams, L1 = L2 = 70 dB SPL) for WT controls (black) and for *Tecta^Y1870C/+^*mice plotted as two groups: those with (red lines) and those without SOAEs (blue lines). The average (±SEM) noise floor is in gray. ***B***, Difference in dB between the mutants with and without SOAEs and the WT controls. A horizontal line is appended to emphasize that animals with DPOAE reductions greater than ∼25 dB do not produce SOAEs. ***C***, ***D***, DPOAE input–output functions. Results for the *Tecta^Y1870C/+^*mice are plotted in the following two groups: those with SOAEs (red) and those without SOAEs (blue). The average (±SEM) growth functions for WT controls are in black. ***C***, f2 = 12 kHz; ***D***, f2 = 27 kHz.

Because some heterozygotes did not produce measurable SOAEs, we recorded DPOAEs at 2f1-f2, the strongest of the intermodulation distortion components, to quantify the degree to which amplification is reduced. Iso-input functions (also known as DP-grams) are provided in Figure 3*A*, where both primaries were presented at 70 dB SPL. For these measurements, the reported parameter was f2 frequency and the frequency ratio (f2/f1) remained constant at 1.2. Although Tecta^Y1870C/+^ mice have reduced DPOAEs, those animals with SOAEs (red) produce larger DPOAEs than the heterozygotes lacking SOAEs (blue). A two-way ANOVA revealed that the WT controls and the two groups of mutant mice were statistically different from one another (genotype main effect, *F*_(2,56.7)_ = 219.34). The effect of frequency was also significant (frequency main effect, *F*_(19,1076)_ = 115.20), as were the interactions between genotype and frequency (*F*_(38,1076)_ = 5.53). Bonferroni-corrected *post hoc t* tests (corrected α level, *p* < 0.003) also revealed that DPOAE magnitudes were statistically different at several f2 frequencies between WT and *Tecta^Y1870C/+^*mice with SOAEs (5.9–15.5 and 20.5–47.0 kHz), between WT and *Tecta^Y1870C/+^*mice without SOAEs (4.5–47.0 kHz), and between *Tecta^Y1870C/+^*mice with and without SOAEs (5.9, 8.9–20.5, and 35.6 kHz).

The decibel differences between *Tecta^Y1870C/+^*mice with or without SOAEs versus WT mice is also highlighted in Figure 3*B*. These functions show the largest difference relative to controls in the mid-frequency region where DPOAEs are the largest. However, heterozygotes lacking SOAEs produce DPOAEs that are reduced to a much greater degree when compared with WT controls. The horizontal line at −25 dB indicates that SOAEs can be recorded in mice with loss of sensitivity but only when reductions in DPOAE level are less than ∼25 dB. Although DP-grams were also collected at a lower level (L1 = 50 dB; L2 = 35 dB; data not shown), these responses were generally in the noise floor for all heterozygotes independent of SOAE generation.

To obtain “thresholds,” growth functions (also known as input–output functions) were recorded (Fig. 3*C*,*D*) and used to obtain a measure of sensitivity by defining DPOAE threshold as the level of f1 that generates a DPOAE of 0 dB SPL (Maison et al., 2010). As in Figure 3*A*, results are provided for *Tecta* ^+/+^ mice (Fig. 3*C*,*D*, black) and for *Tecta^Y1870C/+^*mice with (Fig. 3*C*,*D*, red) or without SOAEs (Fig. 3*C*,*D*, blue). The input–output functions show threshold shifts for two primary pairs (i.e., f2 = 12 and 27 kHz). These results are presented as two groups. At f2 = 12 kHz, heterozygous mice with SOAEs have an average DPOAE threshold shift of 22 dB, while those without SOAEs are shifted by 32 dB. Only those *Tecta^Y1870C/+^*mice with the largest DPOAEs at 2f1-f2, and the smallest threshold shifts, produce SOAEs. In fact, the incidence of SOAEs would have been higher if heterozygous mice with very small DPOAEs, and thus, presumably, having a large decrease in cochlear gain, were not included. A two-way ANOVA showed a genotype main effect (*F*_(2,45.8)_ = 104.3), a level main effect (*F*_(11,504.1)_ = 142.2), as well as interactions between mouse genotype and level (*F*_(22,504.2)_ = 9.3). Subsequent Bonferroni-corrected *post hoc t*-test (corrected α level, *p* < 0.004) also indicated that WT animals were statistically different from *Tecta^Y1870C/+^*mice with SOAEs for L1 between 30 and 75 dB SPL, and at 90 dB SPL. Controls were also different from *Tecta^Y1870C/+^*mice without SOAEs for L1 between 35 and 90 dB SPL. Finally, the mutant mice with and without SOAEs were statistically different for L1 between 55 and 80 dB SPL. At f2 = 27 kHz (Fig. 3*D*), threshold shifts increased relative to those at f2 = 12 kHz, but primarily for those heterozygotes with SOAEs. The two-way ANOVA revealed a mouse genotype main effect (*F*_(2,37.3)_ = 72.1), a level main effect (*F*_(9,335.4)_ = 80.2), as well as interactions between genotype and level (*F*_(18,335.4)_ = 5.7). Subsequent Bonferroni-corrected *post hoc t* tests (corrected α level, *p* < 0.005) also indicated that WT animals were statistically different from *Tecta^Y1870C/+^*mice with SOAEs for L1 between 40 and 80 dB SPL, and at 90 dB SPL. Controls were also different from *Tecta^Y1870C/+^*mice without SOAEs for L1 between 50 and 90 dB SPL. There was, however, no statistically significant difference between the two groups of mutant mice at any stimulus level for f2 = 27 kHz.

It is unlikely that a difference in age between the two groups of heterozygotes, those with and those without SOAEs, underlies the degree to which they produce SOAEs, as mice with missense mutations in *Tecta* are not known to have progressive hearing loss, at least during the first 5 months of life (Legan et al., 2014). In addition, the heterozygous mice without SOAEs were younger (28.4 d of age) than heterozygotes with SOAEs (33.1 d of age) and were similar in age to their WT controls (29.7 d of age). We also wish to emphasize that the control data for this strain are similar to previous results obtained for all of the controls used in our previous work on various TM mutants. Specifically, the average (±SEM) DPOAE threshold for the controls in this study at f2 = 12 kHz was 37.5 ± 0.8 dB SPL (*n* = 16), while that for the *Ceacam^+/+^* controls on a B6 background was 37.2 ± 0.6 dB SPL (*n* = 21; Student’s *t* test, *p* = 0.72). At f2 = 27 kHz, the average (±SEM) WT DPOAE threshold in this report was 45.4 ± 2.0 dB SPL (*n* = 11), while that for the *Ceacam^+/+^* controls was 45.0 ± 1.5 dB SPL (*n* = 11; Student’s *t* test, *p* = 0.87).

In contrast to the DPOAEs, thresholds obtained using ABRs were shifted by ∼50 dB at all frequencies for all mutants, independent of whether they produced SOAEs or not. This observation is consistent with previous measurements of compound action potential thresholds in this mutant (Legan et al., 2005). For example, WT ABR thresholds (*n* = 8) at 12 kHz were 18.3 ± 1.2 dB SPL (mean ± SEM), while those for the *Tecta^Y1870C/+^*animals (*n* = 15) were 67.8 ± 1.5 dB SPL, with an average threshold shift of 49.5 dB. At 27 kHz, the WT ABR thresholds were 20.7 ± 1.5 dB SPL, while those for the *Tecta^Y1870C/+^*were 72.5 ± 1.6 dB SPL, resulting in an average threshold shift of 51.8 dB. The greater loss in neural responses relative to the emissions recorded in *Tecta^Y1870C/+^*mice is thought to relate to an increase in the subtectorial space in the region around the IHC stereocilia (Fig. 1*B*), resulting in reduced IHC stimulation (Legan et al., 2005).

### Anatomical changes in *Tecta^Y1870C/+^*mice are location dependent

**Figure 4.**
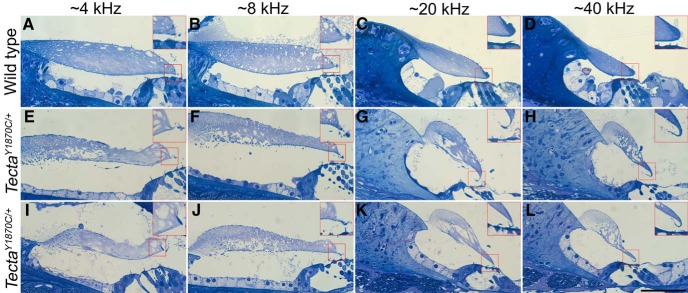
***A–L***, Anatomical changes in the TM. Toluidine blue-stained sections from one WT mouse (***A–D***) and two *Tecta^Y1870C/+^*mice (***E–H***, ***I–L***) at locations along the cochlear spiral associated with regions coding ∼4, 8, 20, and 40 kHz. Boxed regions show 2× enlargements of Kimura’s membrane. Scale bar, ***L*** (for ***A–L***), 50 μm.

Given the frequency dependence of the threshold shifts, we re-examined the anatomy since the previous publication (Legan et al., 2005) described the structural changes at a single basal location (Fig. 1*B*, diagrammatic form). Hence, we provide additional information to obtain a better understanding of changes longitudinally along the cochlear spiral. In Figure 4, images from a WT control (Fig. 4*A–D*) and from two *Tecta^Y1870C/+^*mice are shown at four locations corresponding to regions that are associated with ∼4, 8, 20 and 40 kHz, according to the Liberman Laboratory protocol. At all locations, the marginal band is detached, the extent of the limbal attachment zone is reduced and holes appear in the striated-sheet matrix within the core of the TM. In addition, and as reported by Legan et al. (2005), Kimura’s membrane is delaminated at both the 20 and 40 kHz locations in heterozygotes (Fig. 4*G*,*H*,*K*,*L*). The inset in Figure 4*K* also shows that the OHC stereocilia remain connected to the detached Kimura’s membrane. At the more apical locations (4 and 8 kHz; Fig. 4*E*,*F*,*I*,*J*), delamination is not obvious. Since the degree to which Kimura’s membrane is detached from the body of the TM varies along the cochlear partition, this anatomical change could contribute to the variability in threshold shifts among the *Tecta^Y1870C/+^*animals.

### Multimodal SOAEs are observed *Tecta^Y1870C/+^*mice

**Figure 5.**
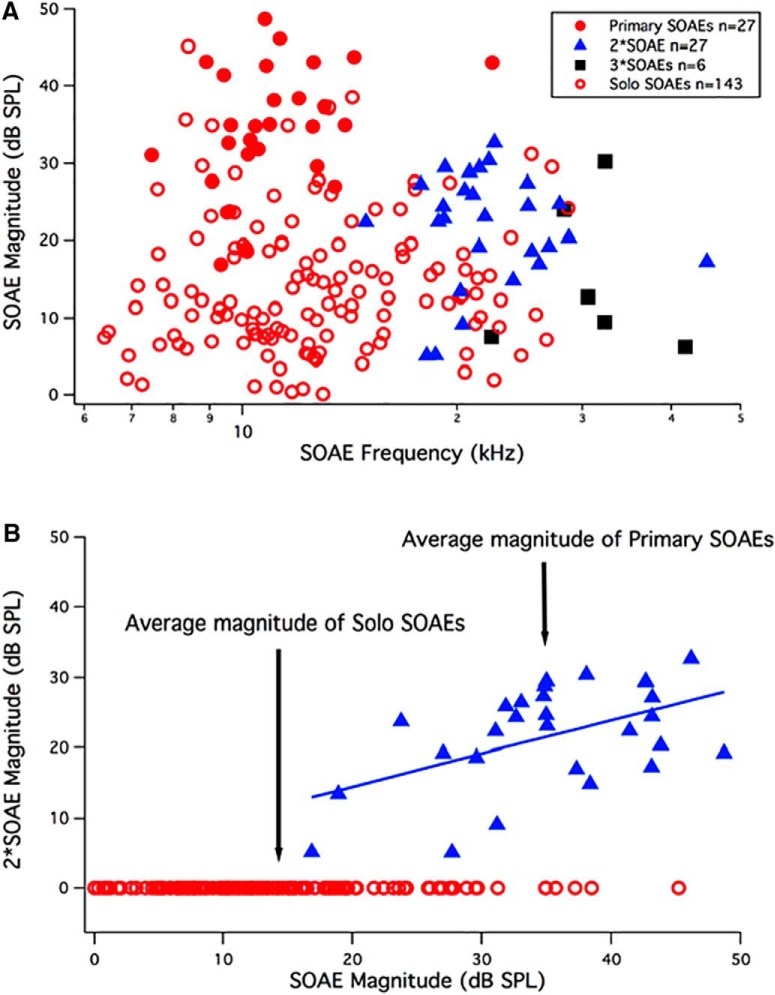
SOAE Magnitudes. ***A***, SOAE magnitude is plotted as a function of SOAE frequency such that primary SOAEs with harmonic partners appear as solid red circles. The second-harmonic SOAEs (2*SOAE) are the blue triangles, and the third harmonic SOAEs (3*SOAE) are the black squares. Solo SOAEs lacking harmonic partners are plotted as red open circles. ***B***, The magnitudes of second-harmonic SOAEs (2*SOAE) are plotted as a function of SOAE magnitude to emphasize that the largest SOAEs are usually associated with second-harmonic partners. The open red circles, arbitrarily plotted at 0 dB SPL, represent solo SOAEs that are not associated with harmonic partners. The blue triangles represent primary SOAEs that were associated with second-harmonic partners. SOAEs <0 dB SPL were not used for analysis. A regression line (SPL_2*SOAE_ = 4.93 + 0.474*SPL_SOAE_) is appended to highlight the relationship between the magnitude of the primary SOAE and that of its second-harmonic partner.

We also examined the relationship between individual emissions and noticed that some SOAE frequencies were integer multiples of a lower frequency SOAE. To distinguish these components, we plotted SOAE magnitude as a function of SOAE frequency for the *Tecta^Y1870C/+^*animals that produced SOAEs (*n* = 32). In Figure 5*A*, SOAEs are plotted as red circles such that “primary” SOAEs associated with higher harmonic/secondary SOAEs appear as filled circles (*n* = 27). SOAEs lacking associated harmonics are designated as “solo” SOAEs (*n* = 143) and appear as open red circles in Figure 5*A*. The blue triangles in Figure 5*A*designate SOAE frequencies that are twice the frequency of a primary SOAE (i.e., second harmonics; 2*SOAE, *n* = 27). The black squares in Figure 5*A*represent SOAEs with frequencies that are three times that of a primary SOAE frequency (3*SOAE, *n* = 6). We will refer to SOAEs that are integer multiples of lower-frequency primary SOAEs as harmonic or secondary SOAEs. The number of SOAEs excluding those that appear to be secondary SOAEs (i.e., excluding both second and third harmonic SOAEs) is 170 (Fig. 5*A*, all red symbols). As a group, primary plus solo SOAEs have an average (±SEM) frequency of 13.5 ± 0.4 kHz and an average magnitude of 18.3 ± 0.9 dB SPL (*n* = 170). Primary SOAEs with harmonic partners (*n* = 27) have a lower average frequency of 11.3 ± 0.5 kHz (Student’s *t* test, *p* < 0.01) but a larger average magnitude of 35.0 ± 1.4 dB SPL (Student’s *t* test, *p* < 0.01). On average (±SEM), the second (third) harmonic SOAE frequencies were 22.7 ± 1.0 kHz (31.3 ± 1.2 kHz), and the magnitudes were 21.5 ± 1.3 dB SPL (15.0 ± 1.8 dB SPL). In addition, the 27 second harmonic SOAEs were on average 13.5 dB down in magnitude from the fundamental primary SOAE and were always lower in level. In contrast, the third harmonics were on average 23.3 dB down.

Harmonics also appear to be associated with the larger SOAEs, as shown in Figure 5*B*, where second-harmonic (2*SOAE) magnitude is plotted as a function of SOAE magnitude. Solo SOAE magnitudes (Fig. 5*B*, open red circles) do not have second-harmonic partners; hence, they are arbitrarily plotted at 0 dB SPL. Primary SOAE magnitudes with harmonic partners are plotted with blue triangles. The average magnitude for primary SOAEs with harmonic partners (35.0 ± 1.4 dB SPL; *n* = 27; Fig. 5*A*, filled red circles) was statistically larger (*p* < 0.001) than for solo SOAEs (14.1 ± 0.01 dB SPL, *n* = 143; Fig. 5*A*, open red circles). In addition, there is a general tendency for larger primaries to associate with larger harmonic partners. In fact, one can fit a linear regression line between second-harmonic and primary magnitudes in the form: SPL_2*SOAE_ = 4.93 + 0.474*SPL_SOAE_. The apparent correlation between primary and secondary SOAE levels (correlation coefficient, *r* = 0.51) suggests that in cases of low-level solo SOAEs, their “secondary” SOAEs may have fallen below the system noise floor.

### Harmonic SOAEs are recorded in regions where DPOAEs and SFOAEs are small or absent

**Figure 6.**
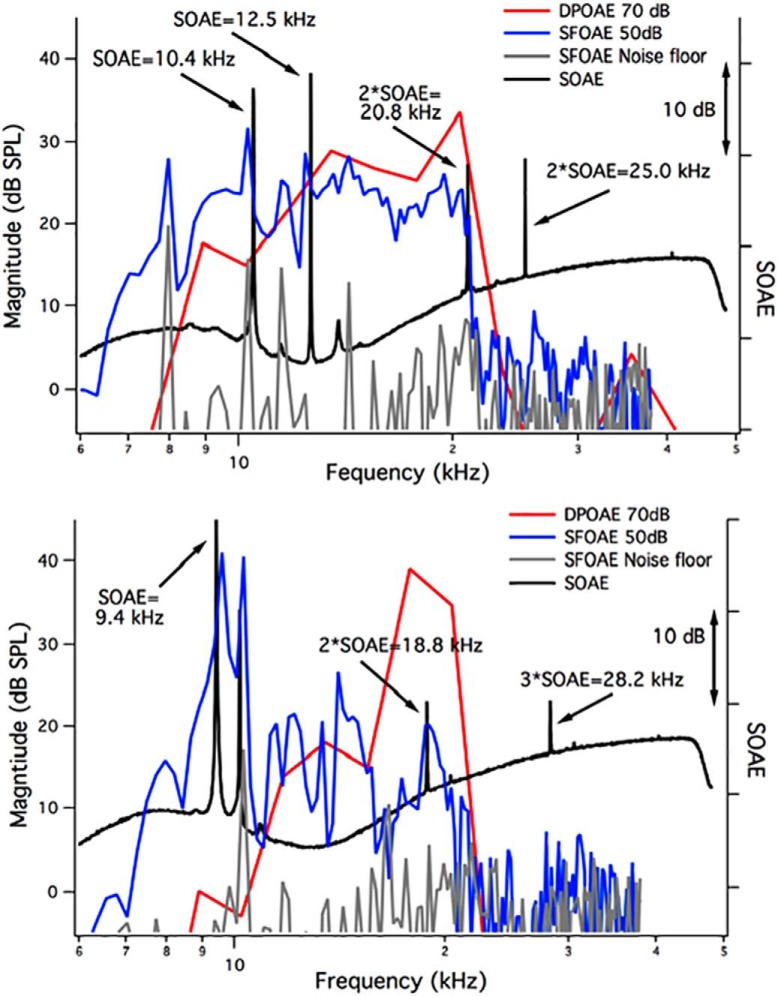
SOAEs, DPOAEs, and SFOAEs in *Tecta^Y1870C/+^*mice. ***A***, ***B***, SOAE spectra (black, right ordinate), individual DPOAEs at 70 dB SPL (red, left ordinate), and individual SFOAEs at 50 dB SPL (blue, left ordinate) are provided for two *Tecta^Y1870C/+^*mice showing primary and secondary/harmonic SOAEs. The SFOAE noise floor is also appended (gray, left ordinate).

In Figure 6, we provide examples of the SOAE spectra (black) in *Tecta^Y1870C/+^*mice plotted along with the individual animal’s iso-input function or DP-gram at 70 dB SPL (red) and their SFOAEs collected at 50 dB SPL (blue), along with the noise floor (gray). In Figure 6*A*, second-harmonic SOAEs (2*SOAE) are associated with primary SOAEs at 10.4 and 12.5 kHz. Notice that the second harmonic at 25.0 kHz lies beyond the high-frequency cutoff of the DP-gram (i.e., at a frequency where amplification is presumably lacking). The high-frequency SFOAEs are also very small or in the noise. In Figure 6*B*, the primary SOAE at 9.4 kHz is associated with both second and third harmonics, with the latter beyond the high-frequency cutoff of the DP-gram and where the SFOAEs are in the noise. This animal is one of only two examples where the third harmonic was comparable in magnitude to the second harmonic. In all other *Tecta^Y1870C/+^*mice (*n* = 19 of a total of 21 animals) with harmonic patterns, the spectra contained only second harmonics. If present, third harmonics were much smaller in magnitude.

### Multimodal SOAEs are not suppressed by nearby external tones

**Figure 7.**
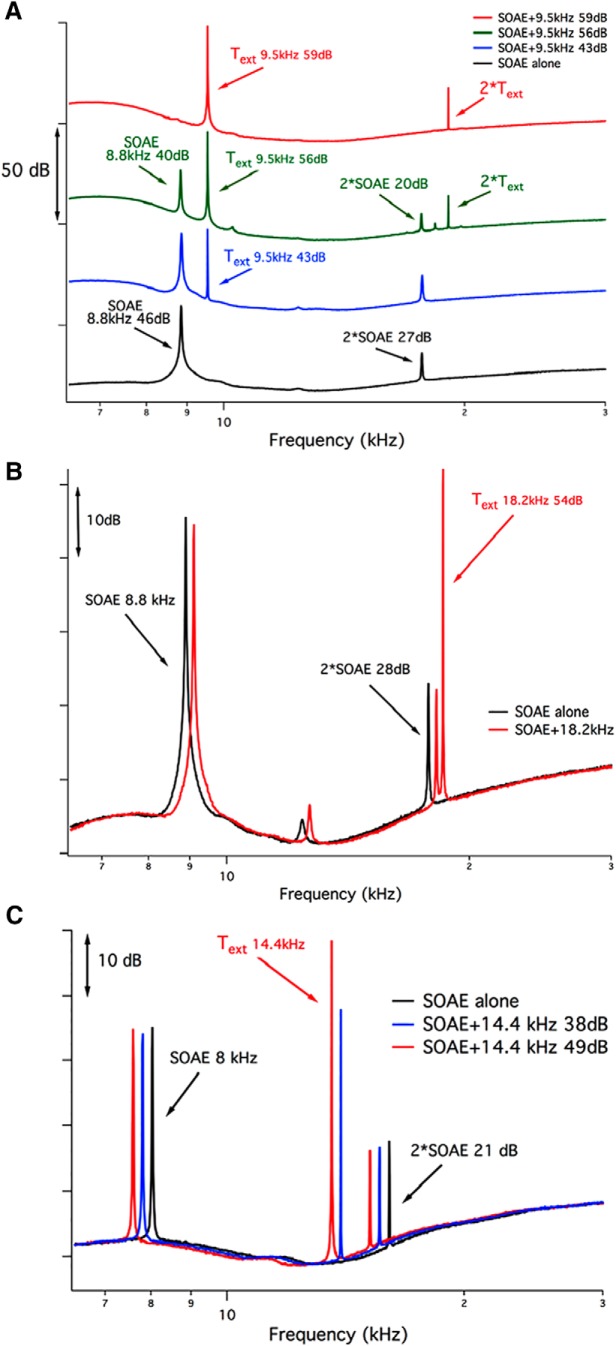
The use of external tones to suppress SOAEs. ***A***, A T_ext_ at 9.5 kHz is introduced just above the primary SOAE at 8.8 kHz (46 dB SPL) and increased in level. The suppressor was not associated with a second harmonic when presented at 43 dB SPL (blue trace), but increasing its level to 56 dB SPL (green trace) and to 59 dB SPL (red trace) generated a 2*T_ext_ component at 19 kHz. Changes did not occur in either primary or secondary SOAEs for T_ext_ = 43 dB SPL but were observed at both 56 and 59 dB SPL. At 59 dB SPL, the suppressor nearly extinguished the primary SOAE, which is now observed as a small ripple in the noise floor. The SOAE-alone condition is plotted in black. To prevent overlap, the SOAE traces have been shifted vertically. ***B***, Results obtained in the same mouse (i.e., the primary SOAE is 8.8 kHz, the second-harmonic SOAE is 17.6 kHz, but the external tone is now 18.2 kHz at 54 dB SPL). For clarity, the SOAE spectrum obtained in the presence of the T_ext_ is shifted horizontally to the right. Minimal changes are observed in the SOAE spectrum when the T_ext_ is presented. Because the SOAEs are plotted on an expanded ordinate in this panel, the SOAE at ∼13 kHz is now visible. This component appeared as a small blip in the waveforms shown in ***A***. ***C***, Data from another *Tecta^Y1870C/+^*mouse where an external tone at 14.4 kHz is introduced at either 38 or 49 dB SPL in an attempt to suppress the second-harmonic SOAE (2*SOAE = 16.0 kHz) associated with the primary SOAE at 8.0 kHz. The SOAE alone is plotted in black, while the SOAEs plus the external tone are in red (T_ext_ = 49 dB SPL) and blue (T_ext_ = 38 dB SPL). Spectra obtained in the presence of the T_ext_ are shifted slightly to the left for clarity.

To study the suppressibility of SOAEs, we introduced external tones (T_ext_s) at various levels and frequencies to determine the degree to which they reduced the magnitudes of either primary and/or secondary SOAEs. In Figure 7*A*, the T_ext_ at 9.5 kHz is just above a primary SOAE at 8.8 kHz (46 dB SPL) that has a second-harmonic SOAE partner at 17.6 kHz (27 dB SPL). For all panels in Figure 7, the SOAE spectra obtained in the absence of a suppressor, the alone conditions, are plotted in black. When the external tone was presented at 43 dB SPL (Fig. 7*A*, blue trace), it had no effect on the primary SOAE or on its second-harmonic partner at 17.6 kHz (2*SOAE). However, at 56 dB SPL (Fig. 7*A*, green trace), the 9.5 kHz suppressor reduced the SOAE and the 2*SOAE by ∼6 dB. When the T_ext_ increased to 59 dB SPL (Fig. 7*A*, red trace), the primary SOAE was seen only as a ripple in the noise floor, and its second-harmonic partner was eliminated. Because the primary SOAEs associated with second-harmonic partners are large, the level of the external tone must be relatively high to produce any reduction. Hence, the suppressor tone also generated a second-harmonic component in the spectrum (2*T_ext_), although no distortion was observed in coupler measurements.

The corollary of this experiment was then performed in the same animal by introducing a T_ext_ at 18.2 kHz, just above the secondary SOAE in frequency, as shown in Figure 7*B*. No changes were observed when the T_ext_ was introduced at 54 dB SPL (i.e., 26 dB SPL larger than the second-harmonic SOAE at 17.6 kHz; Fig. 7*B*, red trace). In this example, the SOAE spectrum with the T_ext_ is shifted horizontally to the right for clarity. A second example appears in Figure 7*C*, where a relatively large SOAE is recorded at 8.0 kHz (37 dB SPL). A higher-frequency emission also appears at twice the frequency of the lower component (i.e., 2*SOAE = 16.0 kHz; 21 dB SPL). In Figure 7*C*, the traces in black show the SOAE recorded alone, while the traces in red and blue were obtained in the presence of a T_ext_ at 14.4 kHz, which is slightly lower in frequency than the second-harmonic SOAE. The SOAE spectra obtained with the T_ext_ are shifted down in frequency (to the left) for clarity. In Figure 7*C*, the T_ext_ at 14.4 kHz failed to reduce the second harmonic when introduced at 38 or 49 dB SPL; in other words, when it was either 17 or 28 dB SPL larger than 2*SOAE. We have used this approach on several ears (*n* = 9) of this type and were never able to reduce the second-harmonic SOAE with an external suppressor tone either slightly higher or lower in frequency, but much larger in magnitude. These results are consistent with the idea that secondary, higher-frequency SOAEs are likely harmonics of lower-frequency SOAEs. In other words, both the lower frequency “fundamental” SOAE and what appears to be its harmonic partner seem to originate from the same place along the cochlear spiral. This possibility is consistent with the observation that several secondary or harmonic SOAEs have frequencies that lie above the high-frequency cutoffs of the DPOAE and SFOAE iso-input functions collected at 70 and 50 dB SPL, respectively (Fig. 6*A*,*B*).

## Discussion

### The TM as a whole stabilizes the active process, while Kimura’s membrane is the subcomponent essential for OHC transduction

The TM is part of an amplifying, mechanical feedback loop in which OHCs function as prestin-actuated motors (Zheng et al., 2000). When the internal dynamics within the feedback loop are altered, loop gain can be maintained, but stability decreases and SOAEs emerge (Cheatham et al., 2014, 2016). In other words, genetically induced changes to TM properties alter the balance among individual components of this tightly coupled feedback system. Although structural changes in the main body of the TM result in an increased propensity to generate SOAEs, with different alterations producing varied phenotypes, delamination of Kimura’s membrane in *Tecta^Y1870C/+^*mice produces the greatest number of and largest SOAEs. This observation suggests that the body of the TM evolved to largely damp out spontaneously generated internal vibrations, and that when its influence is removed, the system becomes oscillatory. It seems that only perturbations, such as changes in TM structure, can reveal how difficult it is to maintain stability in the mammalian cochlea where the feedback-induced gain of the OHCs is ∼50 dB (Ryan and Dallos, 1975; Dallos and Harris, 1978; Liberman et al., 2002).

While the TM is a complex structure, hitherto it has been considered as a single element that influences the dynamic mechanical behavior of the cochlea. In *Tecta^Y1870C/+^*mice, the part of the TM that interacts with OHC stereocilia, Kimura’s membrane, is delaminated from the main body of the TM, allowing us to parcel its role independently from that of the TM proper. For example, alterations in TM anatomy in the *Tecta^Y1870C/+^*mice, and specifically the delamination between Kimura’s membrane and the core of the TM, have several consequences. First, previous work (Legan et al., 2005) and the current work (DPOAEs) indicate that OHC stereocilia remain coupled to Kimura’s membrane such that OHC motility-based feedback remains operational albeit with a reduction in gain, especially at high frequencies. Second, because the main body of the TM in *Tecta^Y1870C/+^*mice has little physical contact with the organ of Corti due to delamination of Kimura’s membrane, the TM can no longer provide its normal load to and mechanical influence on the hair bundles of OHCs (Ó Maoiléidigh et al., 2012; Salvi et al., 2015). As a result, SOAEs are observed similar to other TM mutants where internal oscillations are also detected (Cheatham et al., 2014, 2016). We thus suggest that while the principal function of the TM as a whole is to stabilize the high-gain feedback system, its subcomponent, Kimura’s membrane, by itself, is essential for OHC mechanoelectrical transduction by virtue of its intimate contact with the tips of the OHC stereocilia. In other words, shearing motion between reticular lamina and the accessory structure at the attachment point of the OHC stereocilia can be produced by Kimura’s membrane alone. One may then speculate that the evolutionary need for the main body of the TM is to reduce spontaneous oscillations in the high-gain feedback system of the mammalian cochlea.

The ABR data also support the original report on this mutant (Legan et al., 2005), which showed that close apposition between TM and reticular lamina in the IHC region is essential for the appropriate fluid flow in the subtectorial space required for stimulating IHC stereocilia, which are not attached to the underside of the TM (Dallos et al., 1972). Although OHC-mediated feedback persists in *Tecta^Y1870C/+^*mice, it is reduced, which further decreases the input to IHCs, and their ability to convey information to the CNS. Hence, both compound action potentials (Legan et al., 2005) and ABRs (as reported here) are vastly reduced. The fact that the SOAEs increase when mechanical input to IHCs decreases is again further evidence that IHCs are not the source of these phenomena (Trautwein et al., 1996).

### Multimodal SOAEs do not appear to be spatially separated from their primary SOAE partners in animals with a delaminated TM

The observation of multimodal SOAEs in *Tecta^Y1870C/+^*mice (Figs. 5–7) was not unexpected as human subjects (Burns et al., 1984; Jones et al., 1986; Talmadge et al., 1993; Whitehead et al., 1993) also produce SOAEs identified as cubic difference tones or as integer multiples or harmonics of SOAEs at lower frequencies. Although few studies have addressed this issue, Talmadge et al. (1993) showed that the incidence was low with cubic SOAEs comprising only 3% and harmonic SOAEs only 2% of the 588 SOAEs recorded. In contrast, several emitting *Tecta^Y1870C/+^*mice produce secondary SOAEs that are predominately integer multiples of lower-frequency SOAEs. In fact, 21 of 32 *Tecta^Y1870C/+^*mice with SOAEs showed a harmonic pattern. Although we recorded 27 second harmonics and 6 third harmonics, there were only two examples where the third harmonic was similar in magnitude to the second harmonic. In fact, second-harmonic SOAEs were on average ∼14 dB down from their fundamental SOAE partner, while the third harmonic SOAEs were ∼23 dB down. Our data also indicate that secondary/harmonic SOAEs do not appear to originate from a region of the cochlea that coincides with their harmonic frequency. For example, some of these harmonic SOAEs occur above the high-frequency cutoff of the DPOAE iso-input function and in regions where SFOAEs are small or within the noise. This latter observation is consistent with work in human subjects where primary SOAEs are found in regions where SFOAEs are also recorded (Dewey and Dhar, 2017). Given that secondary SOAEs do not appear to be independent of their primary, the number of SOAEs per cochlea should be revised. When harmonic SOAEs (*n* = 33) are subtracted from the total (*n* = 203), the number of independent SOAEs (*n* = 170) per cochlea drops (i.e., to 170/32 = 5.3). This number, however, is still larger than for any other TM mutant characterized to date, as follows: *Otoa^EGFP/EGFP^* = 3.4 (Cheatham et al., 2016); *Tectb^−/−^* = 1.2 (Cheatham et al., 2018); and *Ceacam16^βgal/βgal^* = 2.4 (Cheatham et al., 2014). On average, wild-type animals generate 2.1 SOAEs per cochlea.

Studies on bullfrog sacculus have also demonstrated multimodal SOAEs at 2:1 and 3:1 ratios of SOAE frequencies (Zhang et al., 2015). Although Zhang et al. (2015) report harmonic but not cubic SOAEs, this may reflect the fact that the overlying otolithic membrane was digested, with the result that the hair bundle was free standing. In this condition, there is no bias on the stereocilia in the bullfrog sacculus preparation. However, in our *in vivo* experiments on mice, SOAEs are recorded from an intact preparation and any bias on the OHC stereociliary bundle induced by attachment to Kimura’s membrane should be reflected in the character of the nonlinearity. In animals with normal hearing, the operating point is positioned where the hair cell transducer function has the steepest slope, which is associated with the production of cubic distortion, especially for cochlear locations with high characteristic frequencies (Russell and Sellick, 1978). The reduction in cubic DPOAEs (2f1-f2) and the predominance of second-harmonic SOAEs in some *Tecta^Y1870C/+^*mice implies that the nonlinearity becomes more even order or quadratic in nature. This observation suggests that the mechanical influence of the TM on the OHC stereocilia in *Tecta^Y1870C/+^*mice is altered such that the set point changes and the nonlinearity becomes more quadratic in character, as discussed previously (Frank and Kössl, 1997; Lukashkin and Russell, 1998). In fact, the degree of separation of Kimura’s membrane from the main body of the tectorial membrane (Legan et al., 2005), and the probable elimination of any influence by a second traveling wave in the main body of the TM (Hubbard, 1993; Ghaffari et al., 2007, 2010; Sellon et al., 2014, 2017; Jones et al., 2015), could affect the set point and subsequently reduce sensitivity.

Our use of a T_ext_ to suppress SOAEs (Fig. 7) also has implications for cochlear mechanics and how it changes with alterations to the TM. When using a suppressor that was 13 dB greater in magnitude and just above the primary SOAE in frequency, both primary and second-harmonic SOAEs were decreased/eliminated. This latter result is consistent with work on human subjects where SOAEs could be reduced when the suppressor was higher in frequency and 10-20 dB SPL higher in level than the SOAE (Harris and Glattke, 1992). In contrast, we have never been able to reduce harmonic SOAEs by adding a nearby external suppressor tone even when it was as much as 28 dB larger in magnitude. The failure of nearby suppressors to decrease second-harmonic SOAEs implies that these components do not have their own traveling waves. In other words, a second-harmonic SOAE does not induce basilar membrane displacements at its best-frequency place. It should be emphasized, however, that the morphology of the TM in *Tecta^Y1870C/+^*mice is altered such that Kimura’s membrane separates from the main body of the accessory structure in the region that coincides with the second-harmonic SOAE frequency but probably not with that of the primary SOAE (Fig. 4). This observation suggests that loss of the TM traveling wave and/or its interaction with the BM traveling wave may contribute to the results shown in Figure 7.

### SOAE production requires some degree of amplification

The data reported here for *Tecta^Y1870C/+^*mice also demonstrate that some amplification is required for SOAE production. Similar to the earlier report on *Otoa^EGFP/EGFP^* mice (Cheatham et al., 2016) and the data on human subjects (Probst et al., 1991), these additional results confirm that SOAEs do not present in the ear canal if DPOAE threshold shifts are greater than ∼25 dB. In addition, animals lacking prestin, and hence amplification, as well as other TM mutants with DPOAE threshold shifts of more than ∼25 dB, do not produce SOAEs (M. A. Cheatham, unpublished observations). The fact that some degree of amplification-based sensitivity must be retained to generate large/numerous SOAEs is consistent with the notion that SOAEs reflect the active cochlear processes associated with OHC function. Our present results, and those published previously (Legan et al., 2000, 2005, 2014; Russell et al., 2007; Lukashkin et al., 2012; Kammerer et al., 2012; Cheatham et al., 2014, 2016, 2018), also indicate that each TM mutant presents its own individual collection of anatomical and consequent physiological changes. In other words, there is no simple correlation between a particular change in TM structure and the change in propensity to generate SOAEs, particularly their magnitude and spectra. Although Hensen’s stripe is missing, or detached and fragmented, in all mutants tested to date, this structure is prominent in WT mice, but only in the basal half of the cochlea. This longitudinal variation makes it difficult to understand how loss of this structure, by itself, would foster the expression of SOAEs with frequencies as low as 5 kHz. If the loss of Hensen’s stripe were in fact the anatomical correlate of this behavior, SOAEs observed in emitting WT controls should appear at low and not at the high frequencies where they have been observed (average SOAE frequency, 22.3 kHz).

Because various alterations in TM anatomy can ultimately result in similar changes to its internal dynamics, measuring SOAEs provides insights into the interplay between the intrinsic properties of the OHC stereocilia and the mechanical influence exerted by the TM *in vivo* (Ó Maoiléidigh et al., 2012; Salvi et al., 2015). In thinking about these processes, it is of interest that homeostasis, a process designed to preserve internal constancy using control systems stabilized by feedback loops, is now being incorporated into computer simulations of cochlear function (Milewski et al., 2017). Based on our work in mutant mice, the TM is very much involved in the maintenance of homeostasis (functional equilibrium) and in stabilizing a feedback network, the integrity of which is required for the sensitivity, selectivity, and dynamic range of cochlear responses, the hallmarks of mammalian hearing.

## Synthesis

Reviewing Editor: Leonard Maler, University of Ottawa Decisions are customarily a result of the Reviewing Editor and the peer reviewers coming together and discussing their recommendations until a consensus is reached. When revisions are invited, a fact-based synthesis statement explaining their decision and outlining what is needed to prepare a revision will be listed below. The following reviewer(s) agreed to reveal their identity: Kevin Ohlemiller, k Charaziak.

Editor Summary and Comments

Both reviewers agree that this manuscript is a strong contribution to our understanding of cochlear sensitivity and tuning. The emphasis on otoacoustic emissions and basilar membrane function may also have important implications for the diagnosis of hearing deficits. Both reviews are very thorough and clearly identify areas for improvement. My own reading of the Ms and the reviews is in full agreement. I think that this Ms is potentially a fine contribution to the literature and, if the necessary revisions are made, acceptable for eNeuro. The revised Ms must be re-reviewed by both reviewers before a final decision can be taken.

Reviewer #1

This is a very engaging and significant paper dealing with essential aspects of mammalian cochlear sensitivity and sharp tuning. The primary findings appear sound and the authors' assertions are reasonable. There are a number of problems with the presentation, as I list below. 

Lines 15-17. The use of ‘nearby’ and ‘regions’ is confusing here, as one deals with frequency and the other is spatial. Please reword. 

Line 30. Should be ‘...(OHCs), which are required...’. 

Lines 44-50. Please break up run-on sentence. 

Line 56. ‘...TM, which extends...’ The referent of ‘which’ here is TM. This sentence is confusing. 

Line 73. Delete comma. 

Line 83. ‘wildtype’ here is meaningless, since there is no strain reference. There are over 400 inbred strains. One cannot 

say that, across 400+ ‘wildtype’ strains, only 6% are spontaneous emitters. In general, avoid making broad claims about mice based on one or a few strains. Lines 182-184 have the same problem. 

Lines 85-91. The topic changes from Ceacam16 to Otoancorin, and then back. This is confusing. 

Line 91. Please clarify at what ages do Ceacam16 KO mice hear normally or abnormally. 

There are multiple issues with ABR thresholds. First, it is disappointing that the reader must find another paper to view thresholds in these mice. The minimal treatment given here (no graph, 2 frequencies) leaves too many questions in the readers' mind. In addition, since these animals carry their own masking noise, it is important to separate threshold shifts due to cochlear anatomy versus shifts due to masking. Please address this. 

Animals Section (line 101). The description of the animals is insufficient. B6129F1/J is not a strain sold by JAX, and can only be produced by a single (first fillial) mating (at JAX) of two strains. If breeding was HetxHet, then the parental strains must have both been Hets for the Tecta allele. We are not told if the parentals were inbred, nor are we told which 129 strain was used. May of these have threshold problems, which raises doubts in the reader's mind about the hearing of the parents. The wild type specimens shown in Figure 4 have abnormal inner sulcus cells (merits a comment, so one may wonder how normal they are. 

Lines 104-106. Please move stats methods to its own section. 

Line 114. What kind of microphone? 

Lines 115-137. The text here is disordered. The methods rove from SOAEs to DPs and back, so that the reader cannot tell what methods or software was used. Please describe each method in its entirety without switching topics. What software was used to record SOAEs? The lack of a complete description here forces the authors to introduce methods details elsewhere that could be more succinctly described here. (e.g., DPOAE L1 and L2 for DP-grams) 

Line 187. Suggest ‘...SOAEs. Some approach...’ for emphasis. 

Line 191. ‘6.3 SOAEs per ear’. This is confusing. The authors have not addressed the temporal aspect of the SFOAEs, so that this could be interpreted as the average over time. When there are that many (if all simultaneous and invariant), does it include two primaries and their harmonics? Line 257 has the same problem. Please clarify. 

Figure 3b is poorly described and not really helpful. Also, ‘SPL’ should be deleted from the Y-axis label, since differences in dB SPL levels are shown. I really suggest 3B be deleted. 

The point made in lines 207-208 (about the significance of threshold shifts less than 25 dB) could be better shown using some other kind of graph. Lines 207 and 208 also refer to sensitivity and thresholds. DPgrams do not deal with sensitivity or thresholds. 

There are no stats in Figure 3. At least the data of Fig. 3A should be subjected to 2-way ANOVA for overall differences. 

The data in Fig. 3D at 70 dB don't match the data in 3A. 

Lines 220-222. Would be clearer to show this in a graph. 

Line 235. ‘greater loss’. Compared to what? 

Lines 237-241. Suggest move to Discussion. 

Line 286. Suggest ‘harmonic, with’. 

The Discussion is long for this article. Addition of headings would help the flow. 

Could the authors please comment on the mechanical nature of the SOAEs. What is going into oscillation? The hair bundle, the OHC soma, or both? 

Line 357. Suggest ‘similar, but to’. 

Lines 373-375. What happened here? 

The two sentences in lines 412-416 are very similar and could be condensed. 

The histopath is an important part of the paper. Even though this is an e-journal, formatting still matters. Figure 4 should be rendered as two columns so that it will not be reduced as much in the final paper. 

Reviewer #2

The manuscript describes measurements of otoacoustic emissions (OAEs) in a mutant mice (Tecta). This particular mutation affects the structures associated with the tectorial membrane (TM) particularly so at the basal end of the cochlea. This report focuses on the description of spontaneous (S)OAEs although other types of OAEs are mentioned as well. The mechanical and neural responses of these mutants at the basal end of the cochlea have been described previously (Legan et al., 2005) thus it is of value to expand on these data by including more comprehensive tests of OAEs. The Authors focus on SOAEs has been motivated by previous studies, where increased prevalence of SOAEs has been observed in mice with mutations targeting TM (Ceacam16, Otoa). Thus, the current report is of value to scientific community concerned with cochlear (micro)mechanics and its relation and reflection in SOAEs. On the other hand, the report investigation of SOAE suppressibility is incomplete and difficult to interpret. This part of the study requires either additional data or major revision of the drawn conclusions. The concept of this part of the study is very interesting and it would be of high value if the Authors repeat some of the experiments on other set of Tecta mutants in a more systematic way. The most crucial part missing is an experiment showing that external tone is capable of suppressing the “primary SOAE”. This is crucial to a) show that there are no paradigm/technical issues with the experiment (such suppression is expected; see below); b) to support the proposed idea that the harmonic SOAE indeed depend on the primary SOAE (reduction in the primary SOAE should result in reduction of the harmonic SOAEs). SOAE suppression has been relatively well documented in humans (reviewed in Harris and Glattke, 1992) where it has been shown that SOAEs are best suppressed by external tones of slightly higher frequencies and higher in level (+10-20 dB for 3-dB suppression) that the SOAE itself. While I would expect that tones presented in this study should produce some noticeable suppression of the harmonic SOAEs, there is little known on OAE suppression in mice in general, thus it is not known whether such external tones were indeed sufficient to produce SOAE reduction. From these reasons I find that part of the study lacking and data being insufficient to support the conclusion that the secondary SOAE indeed originates as harmonic of the primary SOAE. Unequivocally showing that these harmonics SOAEs cannot be suppressed (unlike the primary SOAE) has very important implications from cochlear mechanics point of view: the harmonic SOAEs appear not to vibrate the basilar membrane (BM) near their own best place! This is extremely important if one asks the question how these harmonic SOAEs actually propagate within the cochlea (if these were traveling waves on the BM they should excite their own tonotopic place). Thus, this part of the study can have crucial implications in the world of cochlear mechanics and OAE generation if conducted in a systematic way. 

Specific comments by line number: 

2: ..(SOAEs) This has been a common typo: when a plural form is abbreviated it should be reflected in the abbreviation as well 

2: signals recorded in the ear canal 

3: outer-hair-cell 

5: please use “wild-type”¬ instead of “normal” 

6: incidence of SOAEs 

16: stimulus-evoked 

17: whether they cannot exist independent of their primary it has not been shown in this study. The suggested experiment with an external tone played near the primary resulting in suppression of the harmonic SOAE would support such notion. The fact that these harmonic SOAEs were not suppressed (at least for a limited set of external tone conditions here) and did not coincide with large evoked OAEs is very curious and suggests that these SOAEs do not vibrate their own BF place on the BM. 

29: (OAEs) 

30: (OHCs) 

32: (SOAEs) 

33: please use “wild-type”¬ instead of “normal” 

34: ... to which.. 

40: (IHCs) 

42: (OHCs) 

44: add reference for gain control of OHCs through MOC 

63: E.g.: Kimura showed that the tallest OHC stereocilia are inserted into this part of the TM, and thus this region has been referred to as Kimura's membrane. 

69: large shifts in hearing thresholds OR hearing loss in present across frequencies. 

79: perhaps mention that large thresholds shifts were observed in CAPs not so much in DPOAEs in the report by (Legan et al., 2005) (e.g., as thresholds are mentioned in lines 69-70). 

81: (SOAEs). Also the OAEs and SOAEs in particular should be introduced somewhere more broadly (e.g., similarly as it has been done in the significance). It would be recommended to mention that SOAEs are and SFOAEs are often considered to originate through similar mechanism (coherent reflection) while DPOAEs typically receive dominant contributions from nonlinear distortion source (Shera and Guinan, 1999). 

82: in the absence of acoustic stimulation 

96: and their wild-type controls 

102: extra ‘(’ 

104: What was the screening procedure? Were there any study inclusionary/exclusionary criteria based on the result of the screening? 

104: Was the screening age same as test age? 

115: Please explain in more detail how calibration was performed and verified or provide reference to a study that used similar procedures. 

116: What was the buffer size? 40 spectral averages is not very informative when the buffer size or time of averaging is not provided (“several minutes” does not sound very precise). 

117: spectrum was smoothed ... into 93-Hz wide windows. What was the resolution of the FFT? This seems very important when establishing whether an SOAE is a harmonic or not of a lower-frequency SOAE. Furthermore it seems better not to smooth the spectra for identification of such harmonics altogether. 

118: 0 dB SPL ? It is a very common omission throughout the manuscript (including figures) to not provide a reference for dBs. Because dB is a relative scale without providing the reference value (e.g., 20uPa which typically is denoted as SPL, or 1V if referenced to microphone voltage) it is basically meaningless. Please also revise lines: 187, 188, 191, 198, 209, 213, 220, 231-234, 274, 276, 282, 284, 318, 585, 603, 604. 

Only when a difference/shift is reported just a ‘dB’ unit can be used (i.e., correct y-axis label on Fig. 3B should be ‘dB’ not ‘dB SPL’) 

119: (DPOAEs) 

120: .. stimulus tones, f1 and f2, with a frequency ratio.. 

121: please provide the step for the f2 change and the L1 and L2 levels. 

129: please add frequency step for SFOAE test. 

135: ‘masking’ is typically used I reference to psychoacoustics. It seems that ‘suppression’ is a more appropriate term to use here. 

137: this seems a little confusing. Was the external tone just measured in the ear canal as SOAE was? 

168-170: that link should be provided as a reference 

179: the criteria (other than level &gt; 0 dB SPL) what was considered as an SOAE should be provided. It is not clear in Fig. 2 what was considered an SOAE in WTs. 

194: DPOAEs (delete “distortion product..” as the abbreviation has been already introduced) 

196: please consider deleting “and located just below f1 in frequency” 

203: the differences would be more intuitive to interpret if plotted re. WT (i.e., mutant minus WT DPOAEs) so the y-axis is negative (i.e., indicating dBs below the WT DPOAE levels). 

207-208: but only when reduction in DPOAE levels are less than ~25 dB 

211: DPOAE thresholds 

218: an average DPOAE threshold shift 

220: consider adding the results of statistical testing to the figure (mark significant differences with *) 

223: Do these mice exhibit any decline in DPOAEs with age? I.e., was the average age of mice in SOAE+ and SOAE- group similar? 

225: at 27 kHz the threshold shifts increased relative to the data at 12 kHz only for SOAE+ group but no SOAE- one. Please revise. In general it appears from the legend in Fig. 3 that not all mice were tested with all tests (varying n numbers between panels). That should be pointed out in the Methods. 

229: ..were shifted by ~50 in all mutants (with or without SOAEs (?)) as compared to the controls, 

270: “i.e.,” used twice, please revise 

277-278: were the SOAE harmonics ALWAYS lower in level as compared to the primary SOAE? 

284: Did you mean SFOAEs? Also I don't find it that informative to plot average SFOAEs. Including SFOAEs measured in that particular animal would be better as it is expected for SOAE to align with SFOAE level peaks. A word of commentary whether there were differences in SFOAEs measured in WT and mutants would be very valuable for the study conclusions. For instance, in CEACAM16 both SOAE and SFOAEs were increased in magnitude consistent with a theory that mutation increased “cochlear roughness” and thus all reflection type OAEs were increased as well. 

308-311: the levels of the external tones (both absolute in dB SPL and relative to the SOAE in dB) should be provided. It is not clear from here that the external tones were actually sufficient in level to suppress anything. Furthermore suppression of SOAEs has never been studied in mice to the best of my knowledge. Thus it is crucial to first demonstrate that the external tone is capable of suppressing the primary SOAE (see general comments). 

314-315: to demonstrate that the secondary SOAE originates at the CF place of the primary SOAE an experiment with external tone placed near primary SOAE should be conducted (see general comments) 

321: consider deleting “As noted in the Introduction” 

342: The largest source of damping in the cochlea is fluid not the TM. An alternative explanation would be that disruption in the TM structure produced alternate points of reflection in the cochlea (Shera, 2003). If so, increased SFOAEs would be expected as in CEACAM16 mutants. 

351: shifts in SFOAE thresholds re. WT SFOAE were not demonstrated in this report 

353-362: Based on Fig. 4 the delamination was present only at the basal end of the cochlea ({greater than or equal to} ~20 kHz). The majority of the SOAEs were discovered between 7-20 kHz range. Whether delamination was present in that region it is not clear. This significantly weakens the conclusion on the role of TM proper and Kimura's membrane on their respective roles in the feedback loop. This should be pointed out in the manuscript. 

360-361: Perhaps that should be rephrased to be more general: what appears to be crucial is that the OHC cilia are coupled to something (in this case Kimura's membrane) so they can detect the displacement of the BM. If the cilia are not attached to anything, they are stimulated by fluid drag which could be not as affective. Hence it appears as the shearing motion between reticular lamina and point of the cilia attachment is crucial for the gain production. 

374: multiple repetitions of Salvi et al reference 

391: ...and when on average SFOAEs are small. Were SFOAE data not available for the mice in Fib 5B and C? It is not clear why individual DPOAEs but average SFOAEs are displayed there. 

393: how much larger? dB range? 

406-407: it may be interesting to point that even though the cilia remain embedded in the Kimura's membrane, the bias might have changed due to lack of load from the TM. An interesting discussion of the effects of change in the operational point of the transducer current on production of cubic and harmonic distortion is provided by (Lukashkin and Russell, 1998). 

411: a great simulation of the 

Fig. 2 y-label axes should list the unit dB SPL/ re 1V etc. The WT SOAEs are hard to notice - perhaps an arrow can be added to point these? 

Fig. 3 C and D x-label should probably read dB SPL 

Fig. 5A,B,C - y axis should probably be labeled with dB SPL 

Fig. 6 y-axis label should be updated dB SPL/ re 1 V ? 

References: 

Harris FP, Glattke TJ (1992) The use of suppression to determine the characteristics of otoacoustic emissions. Seminars in Hearing 13:67-80. 

Legan PK, Lukashkina VA, Goodyear RJ, Lukashkin AN, Verhoeven K, Van Camp G, Russell IJ, Richardson GP (2005) A deafness mutation isolates a second role for the tectorial membrane in hearing. Nat Neurosci 8:1035-1042. 

Lukashkin AN, Russell IJ (1998) A descriptive model of the receptor potential nonlinearities generated by the hair cell mechanoelectrical transducer. J Acoust Soc Am 103:973-980. 

Shera CA (2003) Mammalian spontaneous otoacoustic emissions are amplitude-stabilized cochlear standing waves. J Acoust Soc Am 114:244-262. 

Shera CA, Guinan JJ, Jr. (1999) Evoked otoacoustic emissions arise by two fundamentally different mechanisms: a taxonomy for mammalian OAEs. J Acoust Soc Am 105:782-798.
